# Diet production and utilization of corn fermented protein compared to traditional yeast in healthy adult cats

**DOI:** 10.1093/jas/skad272

**Published:** 2023-08-17

**Authors:** Logan R Kilburn-Kappeler, Chad B Paulk, Charles G Aldrich

**Affiliations:** Department of Grain Science, Kansas State University, Manhattan, KS 66506, USA; Department of Grain Science, Kansas State University, Manhattan, KS 66506, USA; Department of Grain Science, Kansas State University, Manhattan, KS 66506, USA

**Keywords:** feline, nutrient digestibility, palatability, stool quality

## Abstract

The inclusion of yeast in pet food can provide health benefits and increase palatability. Corn fermented protein is a co-product from ethanol production which contains approximately 20% to 25% yeast. The objective of this study was to determine the effects of the yeast in CFP on diet production and utilization when fed to healthy adult cats. The four experimental diets included a control with 15% soybean meal (CON) and diets containing either 3.5% brewer’s dried yeast (BDY), 2.5% brewer’s dried yeast plus 17.5% distillers dried grains with solubles (BDY+DDGS), or 17.5% corn fermented protein (CFP). All treatments except CON were formulated to contain 3.5% yeast. Experimental diets were fed to adult cats (*n* = 11) in an incomplete 4 × 4 replicated Latin square design. Cats were adapted to diet for 9 d followed by a 5-d total fecal collection. Titanium dioxide (0.4%) was added to all diets as an external marker to estimate digestibility. Data were analyzed using a mixed model in SAS (version 9.4, SAS Institute, Inc., Cary, NC) with treatment as a fixed effect and cat and period as random effects. Preconditioner discharge temperature was greater (*P* < 0.05) for CON and BDY (average, 96 °C) compared to BDY + DDGS and CFP (average, 91 °C). Extruder screw speed, die temperature, kibble toughness, and kibble hardness were greatest (*P* < 0.05) for CFP. The bulk density of BDY + DDGS at 392 g/L was greater (*P* < 0.05) than BDY and CFP (average, 342 g/L). The sectional expansion index of kibble for CFP was greater (*P* < 0.05) than BDY + DDGS and smaller (*P* < 0.05) than CON but similar to BDY. Fecal output was greatest (*P* < 0.05) for cats fed BDY + DDGS. Nutrient digestibility was lowest (*P* < 0.05) for BDY + DDGS. The concentrations of short-chain and branched-chain fatty acids in fecal samples were not altered (*P* > 0.05) by dietary treatment. Cats had no preference (*P* > 0.05) when comparing CON to BDY or BDY + DDGS. However, cats consumed significantly less CFP compared to CON. The significant differences for bulk density, fecal output, and nutrient digestibility among dietary treatments are likely due to a greater fiber effect of DDGS compared to CFP. Therefore, the yeast component in CFP may provide greater kibble expansion and nutrient utilization compared to DDGS when fed to cats.

## Teaser

Corn-fermented protein is a co-product from ethanol production which contains approximately 20% to 25% yeast. Based on the effects of CFP on processing parameters, kibble characteristics, stool quality, nutrient digestibly, and palatability compared to traditional yeast, CFP would be best utilized as a protein or fiber source for pet food.

## Introduction

Many commercial pet foods contain yeast as an ingredient, with brewer’s dried yeast (BDY) as the most common source. Pet food can also contain yeast extract, yeast cell walls, or yeast culture. Yeast was originally included in pet food as a source of B vitamins, but its function has evolved due to the supplementation of B vitamins in pet food today (Beynen, 2019) . In the current market, nutritional yeast supplements claim to control fleas and promote healthy skin and coat (Beynen, 2019). Whereas BDY is often added at a 1% inclusion to improve the palatability of kibble (Swanson and Fahey, 2007). In addition, extracts from yeast cell walls such as mannan-oligosaccharides (MOS) and beta-glucans are reported to support gut and immune health in dogs (Swanson et al., 2002; Pawar et al., 2017; Rummell et al., 2022; Fries-Craft et al., 2023) and cats (Santos et al., 2018; Calabrò et al., 2020).

Brewer’s yeast is the dried product of the slurry that remains after beer and ale fermentation. Typically, beer is derived from malted barley which is fermented slowly at 10% to 20 °C and produced using a batch fermentation process resulting in approximately 6% alcohol. On average BDY contains 41% protein, 3% fat, and 6% ash on a dry matter basis (Swanson and Fahey, 2007). In addition to its use as a palatant, yeast has been reported to be an adequate protein source in dog diets (Martins et al., 2013; Reilly et al., 2021).

Corn fermented protein (CFP), a co-product from ethanol production, could be utilized as a novel yeast ingredient for pet food. Corn fermented protein contains 53% protein, 6% fat, and 3% ash on a dry matter basis (Kilburn-Kappeler et al., 2022). In addition, CFP contains a substantial yeast component at approximately 20% to 25%. In contrast to the brewing industry, ethanol is produced from corn using a rapid continuous fermentation at 35% to 38 °C, yielding 9% to 12% alcohol. As a result, yeast co-products from the brewing and distilling industries differ in nutrient composition and organoleptic properties due to the difference in substrate and processing methods (Swanson and Fahey, 2007). Previous studies have reported CFP to be an acceptable protein source for both dogs and cats when compared to soybean meal and corn gluten meal (Kilburn-Kappeler et al., 2022; Kilburn-Kappeler and Aldrich, 2023; Smith and Aldrich, 2023), but the yeast component has yet to be evaluated in cats. Therefore, the objective of this study was to compare the effects of the yeast in CFP to BDY, a common yeast ingredient in pet food, on diet production and utilization in cats.

## Materials and Methods

The feeding trial was conducted at Kansas State University Veterinary Medicine Complex (Coles Hall) under the Institutional Animal Care and Use Committee (IACUC) #4348 protocol. The palatability trial was conducted at Summit Ridge Farms (Susquehanna, PA) under protocols KSUPALF00720, KSUPALF00820, and KSUPALF00920.

### Diet formulation

Dietary treatments consisted of a control diet containing 15% soybean meal (CON) and experimental diets containing either 3.5% brewer’s dried yeast (BDY), 2.5% brewer’s dried yeast plus 17.5% distillers dried grains with solubles (BDY+DDGS), or 17.5% corn fermented protein (CFP). It was assumed that CFP and DDGS were comprised of 20% and 5.7% yeast, respectively; therefore, all treatments, except CON, were formulated to contain 3.5% yeast. The formulated diets met the AAFCO nutritional requirements for adult cats. Two main dry rations were purchased from a commercial mill (Fairview Mills, Seneca, KS), which contained corn, chicken meal, beet pulp, fish meal, salt, potassium chloride, vitamin and mineral premix, choline chloride, and natural antioxidant. The inclusion of corn and chicken meal varied slightly among base rations while all other ingredients were maintained. One ration was used for CON and CFP treatments while the other was used for BDY and BDY+DDGS treatments in attempt to maintain nutrient composition among all diets. The remaining ingredients, soybean meal (Fairview Mills, Seneca, KS), CFP (POET Bioproducts, Sioux Falls, SD), DDGS (Fairview Mills, Seneca, KS), BDY (Fairview Mills, Seneca, KS), corn starch (Fairview Mills, Seneca, KS), and corn gluten meal (Fairview Mills, Seneca, KS), were added to the base rations during diet production. Soybean meal, corn gluten meal and/or corn starch were added to CON, BDY, and CFP to create similar nutrient profiles among all dietary treatments and to balance a 20% inclusion of experimental ingredients compared to BDY + DDGS. Titanium dioxide (Fairview Mills, Seneca, KS) was also added to all diets at 0.4% to serve as an indigestible marker to estimate apparent total tract nutrient digestibility (Table 1).

**Table 1.** Ingredient composition of feline diets containing yeast and ethanol co-products on an as-is basis

	Treatment^1^			
Ingredient, %	CON	BDY	BDY+DDGS	CFP
Corn	34.6	30.0	30.0	34.6
Chicken meal	30.0	35.0	35.0	30.0
Soybean meal	15.0	8.0	–	–
Distillers dried grains with solubles	–	–	17.5	–
Corn fermented protein	–	–	–	17.5
Brewer’s dried yeast	–	3.5	2.5	–
Corn starch	–	6.5	–	2.5
Corn gluten meal	5.0	2.0	–	–
Chicken fat	6.0	5.6	5.6	6.0
Other^2^	9.4	9.4	9.4	9.4

^1^CON, control; BDY, brewer’s dried yeast; BDY + DDGS, brewer’s dried yeast and distillers dried grains with solubles; CFP, corn fermented protein.

^2^Other ingredients: beet pulp, fish meal, flavor, titanium dioxide, salt, potassium chloride, vitamin and mineral premix, choline chloride, natural antioxidant.

### Diet production

Each diet was produced using a single screw extruder (model E525, ExtruTech, Inc., Sabetha, KS). The preconditioner (model ADP 145, ExtruTech, Inc., Sabetha, KS) was configured with 12, 45° back and 57 neutral beaters on each of the two shafts. The extruder profile and barrel temperatures were based on a typical commercial pet food configuration. At the end of the extruder barrel, there were two round die inserts with an interior diameter of 3 mm. Dry matrix feed rate (318 kg/h) and pre-conditioner (PC) cylinder speed (185 rpm) were kept constant during the processing of all treatments.

During processing, PC and extruder (EX) parameters were collected from sensor readouts every 2 min to evaluate potential effects of CFP inclusion on the process. Output variables included PC discharge temperature, EX motor load, EX die temperature, total mass flow (TMF), specific mechanical energy (SME), and in-barrel moisture content (MC).

The TMF was calculated by adding the dry feed rate with water and steam injected in PC and EX, assuming that 80% of the water coming from the PC and EX steam is lost during flash-off as kibbles exit the die:

TMF = dry feed rate + PC water + (0.2 * PC steam) + EX water + (0.2 * EX steam)

SME was calculated using the following formula:


$$\begin{array}{l} SME\;\left(\frac{kJ}{kg}\right)=\;\frac{\frac{\tau -\tau_{o}}{100}*\left(\frac{N}{N_{r}}\right)*P_{r}}{m} \end{array}$$

where τ is the EX % torque or EX motor load, τ_0_ is the EX no load % torque (25% at EX screw speed 425 rpm), *N* is the EX screw speed (rpm), *N*_*r*_ is the rated EX screw speed (425 rpm), *P*_*r*_ is the rated EX motor power (114 kW), and *m* is TMF (kg/s).

The in-barrel moisture content (MC) was also calculated using the following formula:


$$\begin{array}{l} MC=\frac{m_{f}\times X_{f}+m_{ps}+m_{pw}+m_{es}+m_{ew}}{m_{f}+m_{ps}+m_{pw}+m_{es}+m_{ew}} \end{array}$$

where *m*_*f*_ is the feed rate, *X*_*f*_ is the moisture content of the raw material, *m*_*ps*_ is the percentage of added steam in the preconditioner, *m*_*pw*_ is the percentage of added water in the preconditioner, *m*_*es*_ is the percentage of steam added into the extruder, and *m*_*ew*_ is the percentage of water added into the extruder. A moisture content of 10% was assumed for *X*_*f*_.

After extrusion, kibble was pneumatically conveyed through an 8″ clean air hood system and deposited onto an oscillating belt spreader. The kibble was dried on a 1.5 m wide single pass two zone dryer (model AFI, ExtruTech, Sabetha, KS) to achieve a less than 10% moisture content. Kibble was dried at approximately 105 °C for 21 min. Dried kibble was coated with chicken fat protected with natural antioxidants (Nutrios, Springfield, MO) and a dry powdered flavor designed for cats (AFB International, St. Charles, MO). Coated diets were stored in poly-lined Kraft paper bags in a warehouse for 3 months prior to feeding.

### Physical characteristics of kibble

Bulk density was measured off the dryer every 15 min during the processing of each treatment. Bulk density was measured using a 1 liter cup in which kibble was leveled and weighed on a digital scale with 0.1 g sensitivity. In addition, every 15 min five randomly selected kibbles from each diet production off the dryer were measured for diameter and length using a digital caliper. Ten randomly selected kibbles off the dryer were also weighed using a digital scale with 0.0001 g sensitivity (EX324N; Ohaus Corporation, Parsippany, NJ, USA). The diameter, length, and mass measurements were used to determine sectional expansion index and specific length.

Sectional expansion index (SEI) was determined by comparing the squared diameter of the dried extruded kibbles with the squared die diameter of the extruder:


$$\begin{array}{l} SEI=\frac{D^{2}}{d^{2}} \end{array}$$

where *D* is the extrudate diameter and *d* is the extruder die diameter.

Specific length in mm/g was determined by the following equation:


$$\begin{array}{l} Specif\;\;ic\;length=\frac{l}{m} \end{array}$$

where *l* is the extrudate length and *m* is the extrudate mass.

A texture analyzer (model TA-XT2, Texture Technology Corp., Scarsdale, NJ) with a 30 kg load cell was used to measure kibble texture. A cylindrical probe (25 mm diameter) was used to compress 30 kibbles within each treatment. The procedure was adapted from Dogan and Kokini (2007) with a test speed of 2 mm/s and a strain level set at 80%. Kibble hardness was considered to be the peak force in kg of the first major kibble breakage and the energy to compress the kibbles to 80% was computed as the area under the curve in kg mm for each compressed kibble not accounting for the negative values. The compression energy was considered as kibble toughness.

### Feeding trial

Eleven healthy adult (3.1 ± 1.7 yr) American shorthair cats (10 males and 1 female) were enrolled in an incomplete 4 × 4 triplicated Latin square design comprised of four experimental treatments and four 14 d periods. Each of the four periods was composed of 9 d for diet adaptation followed by 5 d of fecal collection, which has been reported to be more than sufficient for stabilizing the gut to dietary intervention (Lin et al., 2022). Cats had an average body weight of 5.6 ± 1.7 kg. The daily metabolizable energy requirement was calculated for lean cats with 100 * BW_kg_^0.67^ (NRC, 2006). The food allowance of individual cats was adjusted during each adaptation period if needed to maintain body weight.

The cats were housed on a 12 h light cycle with lights off from 1900 to 0700. During the adaptation period, the cats were group-housed but fed individually. Whereas in the collection period, the cats were individually housed in stainless steel cages. The cats received two feedings per day at 0800 and 1700 h with access to food for 1 h and water ad libitum. During the collection period, all feces and orts were collected daily. All feces were weighed and scored on a 1 to 5 scale in 0.5 increments with a score of 3.5 to 4.0 considered ideal [1 − liquid diarrhea to 5 − dry hard pellets; (Carciofi et al., 2008)]. All feces were scored by a single person for consistency. However, this individual was not blinded to treatments. Feces were stored in labeled whirl-pak bags in a freezer until further processed. In addition, pH of a fresh sample (within 15 min of defecation) was recorded in triplicate with a calibrated glass-electrode pH probe (FC240B, Hanna Instruments, Smithfield, RI), and 2-g aliquots were transferred into three plastic microcentrifuge tubes using a spatula and stored at −80 °C for short-chain fatty acid (SCFA) and branched-chain fatty acid (BCFA) analysis.

### Digestibility calculations

After each collection period, all feces from each cat were composited and dried at 55 °C in a forced air oven until constant weight (24 to 48 h). Dried samples were ground to pass through a 1 mm screen in a laboratory fixed blade impact mill (ZM 200, Retsch, Verder Scientific, Haan, ­Germany). Titanium dioxide (TiO_2_) concentration was measured in food and feces using a spectrophotometric plate reader (Gen5TM, Biotek Instruments, Inc.Winooski, VT) at 410 nm (Myers et al., 2004). Apparent total tract digestibility (ATTD) was estimated by TiO_2_ using the following equation:


$$\begin{array}{l} ATTD=\left[1-\frac{\%\;TiO2\;in\;food*\;\%\;nutrient\;in\;feces}{\%\;TiO2\;in\;feces*\;\%\;nutrient\;in\;food}\right]*100\; \end{array}$$

Digestibility was calculated using both the total collection and titanium dioxide methods, which resulted in similar digestibility values and trends. However, the titanium dioxide method resulted in a lower standard error of the mean. Therefore, digestibility values from the titanium dioxide method were selected to report in this manuscript.

### Nutrient analysis

Food and partially dried fecal samples were analyzed in duplicate for moisture (AOAC 930.15), ash (AOAC 942.05), fat by acid hydrolysis and hexane extraction (AOAC 960.39), gross energy (Parr 6200 Calorimeter, Parr Instrument Company, Moline, IL), and total dietary fiber (AOAC 991.43). Crude protein was determined by Dumas combustion (AOAC 990.03) using a nitrogen analyzer (FP928, LECO Corporation, Saint Joseph, MI).

### Fecal Chemical Analysis

Fecal SCFA and BCFA concentrations were determined by gas–liquid chromatography (Erwin et al., 1961) using a capillary column (30 m × 0.25 mm internal diameter; 0.25 µm film thickness; Aligent Technologies, Santa Clara, CA, USA). The system was equipped using helium as a carrier gas with a constant flow rate of 40 cm/s and utilizing a 25:1 split ratio injector with an injection size of 0.5 µL. A flame ionization detector was configured with hydrogen as the makeup gas with a flow rate of 40 mL/min to clarify peak resolution. The detector and injector temperatures were set at 250 °C, and the initial oven temperature was set to 80 °C with a ramp rate of 10 °C/min to 200 °C. The peak area of chromatograms was analyzed using integrative software (GC solution version 2.42.00, Shimadzu, Kyoto, Japan). Concentrations of SCFA (acetate, propionate, and butyrate) and BCFA (isobutyrate, valerate, and isovalerate) were quantified by comparing the sample peak area to a known standard of 10 mM concentration (Volatile Free Acid Mix, Sigma-Aldrich, St. Louis, MO, USA) and correcting for fecal dry matter content.

### Palatability trial

Experimental treatments (BDY, BDY + DDGS, and CFP) were evaluated for palatability vs. the control diet (CON) by cat panels at a commercial kennel (Summit Ridge Farms, Susquehanna, PA). Each experiment was conducted as a split-plate test, in which two stainless steel bowls containing 100 g of food were presented to animals for a total of 4 h. Each comparison trial was repeated for 2 d, with a bowl position switched daily. Twenty animals were fed daily, providing 40 observations for each paired comparison test. Preference was determined based on animals’ first choice and total food consumption. Data from consumption was represented as the following ratio:


$$\begin{array}{l} Intake\;ratio\;=\frac{consumption\;of\;Diet\;A}{total\;consumption\;Diet\;A+Diet\;B} \end{array}$$

### Statistics

Processing and digestibility data were analyzed using a GLIMMIX procedure in SAS (version 9.4, SAS Institute, Inc., Cary, NC, USA). Tukey’s post hoc test was applied for the least-squares means separation, with significance considered at *P* < 0.05. For each diet production, sampling was conducted at evenly spaced intervals which were considered replicates. The digestibility experiment was conducted as an incomplete 4 × 4 replicated Latin square design. Each of the 11 experimental units (cats) was assigned to treatment using the spreadsheet by Kim and Stein (2009). Dietary treatment was the fixed effect and cat and period were the random effects within the model.

In the palatability experiment, the consumption ratio was analyzed using a *t*-test in a two-way ANOVA and the first-choice preference was analyzed using a Chi^2^ test. The 20 cats were considered the experimental units for analysis.

## Results and Discussion

### Extrusion processing and kibble characteristics

Preconditioner (PC) cylinder speed was maintained at 185 rpm for the production of all dietary treatments (Table 2). Steam flow to the PC was greatest (*P* < 0.05) during the production of CON and BDY at an average of 47 kg/h and lowest (*P* < 0.05) for CFP at 37 kg/h. Steam flow for BDY + DDGS was intermediate at 41 kg/h. Preconditioner water flow was greater (*P* < 0.05) during the production of CON and BDY at an average of 57 kg/h compared to BDY + DDGS and CFP at an average of 49 kg/h. Preconditioner discharge temperature was greater (*P* < 0.05) for BDY + DDGS and CFP at 96 °C compared to CON and BDY at 91 °C.

**Table 2.** Processing parameters and physical characteristics of feline diets containing yeast and ethanol co-products

	Treatment^1^					
Parameter	CON	BDY	BDY+DDGS	CFP	SEM	*P*-value
Preconditioner						
Cylinder speed, rpm	185.00	185.00	185.00	185.00	0.000	1.0000
Steam flow, kg/h	47.47^a^	46.97^a^	40.88^b^	37.39^c^	0.660	<0.0001
Water flow, kg/h	57.35^a^	56.40^a^	48.69^b^	48.50^b^	1.936	<0.0001
Discharge temperature, °C	90.44^b^	91.54^b^	96.36^a^	96.40^a^	0.753	<0.0001
Extruder						
Screw speed, rpm	450.00^b^	450.00^b^	438.16^b^	475.00^a^	4.785	<0.0001
Steam flow, kg/h	0.00^c^	9.07^b^	21.05^a^	13.76^a,b^	3.636	<0.0001
Water flow, kg/h	0.00^c^	0.00^c^	8.18^a^	4.38^b^	0.723	<0.0001
Motor load, amps	67.22^a^	64.43^b^	64.06^b^	64.06^b^	0.661	<0.0001
TMF^2^, kg/h	385.02	385.78	387.43	381.28	2.318	0.0989
SME^3^, kJ/kg	123.55^a^	106.11^b^	101.50^b^	111.77^b^	4.387	<0.0001
MC^4^, %	32.30^a,b^	33.43^a,b^	34.25^a^	32.00^b^	0.786	0.0267
Die temperature, °C	110.97^b^	110.76^b^	109.68^b^	114.21^a^	0.807	<0.0001
Knife speed, rpm	1600.00^c^	1681.48^b^	2000.00^a^	2000.00^a^	25.670	<0.0001
Dryer						
Bulk density, g/L	363.07^a,b^	343.13^b^	392.23^a^	339.97^b^	14.760	0.0171
Kibble diameter, mm	6.82^a^	5.31^b^	4.58^c^	5.68^b^	0.253	<0.0001
Kibble length, mm	4.09^b^	4.36^a,b^	3.35^c^	4.47^a^	0.110	<0.0001
Specific length, mm/g	105.76^c^	135.81^b^	150.57^a^	133.31^b^	4.101	<0.0001
SEI^5^, mm^2^/mm^2^	5.32^a^	3.17^b,c^	2.36^c^	3.64^b^	0.345	<0.0001
Hardness, kg	2.43^b^	2.26^b,c^	2.02^c^	2.84^a^	0.136	<0.0001
Toughness, kg mm	8.28^b^	6.59^c^	7.23^b,c^	9.85^a^	0.564	<0.0001

^1^CON, control; BDY, brewer’s dried yeast; BDY + DDGS, brewer’s dried yeast and distillers dried grains with solubles; CFP, corn fermented protein.

^2^TMF, total mass flow.

^3^SME, specific mechanical energy.

^4^MC, in-barrel moisture content.

^5^SEI, sectional expansion index.

^a-c^Means within a row lacking a common superscript letter are different (*P* < 0.05).

Extruder (EX) screw speed was fastest (*P* < 0.05) for CFP at 475 rpm compared to the remaining treatments at an average of 446 rpm (Table 2). Extruder steam flow was greater (*P* < 0.05) for BDY + DDGS at 21 kg/h compared to BDY and CON at 9 and 0 kg/h, respectively. Extruder steam flow for CFP was intermediate BDY + DDGS and BDY at 14 kg/h. Water flow to the EX was greatest (*P* < 0.05) for BDY + DDGS at 8 kg/h and lowest (*P* < 0.05) for CON and BDY at 0 kg/h, with CFP intermediate at 4 kg/h. Extruder motor load was greatest (*P* < 0.05) for CON compared to the remaining treatments. Total mass flow (TMF) was maintained (*P* > 0.05) among dietary treatments at an average of 385 kg/h. Specific mechanical energy (SME) was greater (*P* < 0.05) for CON at 124 kJ/kg compared to the other treatments at an average of 106 kJ/kg. In-barrel moisture content (MC) was greatest (*P* < 0.05) for BDY + DDGS at 34% and lowest (*P* < 0.05) for CFP at 32%, CON and BDY were intermediate at an average of 33%. Die temperature was greatest (*P* < 0.05) for CFP at 114 °C compared to the remaining treatments at an average of 110 °C. Extruder knife speed was fastest (*P* < 0.05) for BDY + DDGS and CFP at 2000 rpm and slowest (*P* < 0.05) for CON at 1600 rpm.

Bulk density was greater (*P* < 0.05) for BDY + DDGS at 392 g/L compared to BDY and CFP at an average of 342 g/L, while CON was intermediate at 363 g/L (Table 2). Kibble diameter was largest (*P* < 0.05) for CON at 6.8 mm and smallest (*P* < 0.05) for BDY + DDGS at 4.6 mm. Kibble diameter for BDY and CFP was intermediate at an average of 5.5 mm. Kibble length was longer (*P* < 0.05) for CFP at 4.5 mm compared to CON and BDY + DDGS at 4.1 and 3.4 mm, respectively. Kibble length of BDY was intermediate CFP and CON at 4.4 mm. Kibble length was smallest (*P* < 0.05) for BDY + DDGS. Specific length of kibble was largest (*P* < 0.05) for BDY+DDGS at 151 mm/g and smallest (*P* < 0.05) for CON at 106 mm/g, BDY and CFP were intermediate at an average of 135 mm/g. Sectional expansion index (SEI) was greatest (*P* < 0.05) for CON. The SEI of CFP was greater (*P* < 0.05) than BDY + DDGS, with BDY intermediate. Kibble hardness and toughness were greatest (*P* < 0.05) for CFP compared to the remaining treatments. In addition, kibble hardness for CON was greater (*P* < 0.05) than BDY+DDGS with BDY intermediate. Whereas kibble toughness for CON was greater (*P* < 0.05) than BDY with BDY+DDGS intermediate.

The increase in PC discharge temperature for CFP compared to BDY was likely due to the fluctuations in input steam and water. The increased EX screw speed, EX steam flow, and EX water flow with CFP compared to BDY was due to the extruder operator and not the dietary matrix. It would be expected that an increase in screw speed would result in more mechanical energy, increasing material cook and expansion (Rokey, 2006). Therefore, the fastest screw speed would be expected to produce the most expanded kibble, which was observed in this study as CFP had the lowest bulk density numerically. However, the SME results were surprising as CON had the greatest SME rather than CFP. Regardless, CON and CFP resulted in similar bulk density. The motor load is related to screw speed, degree of fill, and viscosity of the feed material in the screw channel (De Pilli et al., 2011). Therefore, the comparable motor load for BDY and CFP indicates that varying ingredients did not impact viscosity. It would be expected that an increase in screw speed would decrease barrel fill, decreasing motor load (Unlu and Faller, 2002). However, screw speed did not appear to affect motor load in the current study, which is supported by the consistent TMF among dietary treatments. The differences in MC among dietary treatments were minimal (<2%) and considered to be of no practical importance. The increased die temperature of CFP compared to BDY was likely due to the increased EX screw speed. The increase in die temperature would also be expected to increase product expansion (Shukla et al., 2005). The increase in knife speed was also due to the extruder operator and not a direct effect of the dietary ingredients. The increased knife speed may have resulted in the deceased kibble length in BDY + DDGS. However, the kibble length of CFP was not affected.

A previous study reported that input processing parameters had to be adjusted to produce a similar product for a diet containing CFP compared to diets containing soybean meal (SBM) or corn gluten meal (CGM; Smith and Aldrich, 2023). That study observed that CFP required more PC water input and mechanical energy to result in a similar bulk density to the remaining treatments, which was attributed to the decreased starch content in CFP compared to SBM and CGM (Smith and Aldrich, 2023). Therefore, increasing PC water input and mechanical energy in the extruder barrel promoted gelatinization in the CFP treatment allowing for improved expansion and similar bulk density among dietary treatments. In the current study, the increased screw speed during the production of CFP would result in increased mechanical energy also promoting expansion. Even though CFP did not result in the greatest SME, the increased screw speed could have resulted in the similar SME of CFP to BDY.

Bulk density is a common and reliable quality control measurement used during the production of dry expanded pet food, which provides a quick and tangible measure of how well the product is cooked or expanded (Rokey et al., 2006). Bulk density and expansion have an inverse relationship meaning the lower the bulk density, the more expanded the product. The comparable bulk density, kibble diameter, kibble length, specific length, and SEI of BDY and CFP indicate that the fiber content in CFP did not impact expansion. Whereas BDY + DDGS resulted in the greatest bulk density and smallest kibble diameter, length, and SEI likely due to the heightened dietary fiber content. As fiber is considered a dispersed phase filler during extrusion and is known to limit kibble expansion (Guy, 2001), which has been supported by previous studies (Hsieh et al., 1989, 1991; Monti et al., 2016; Alvarenga et al., 2018 ). Furthermore, Chevanan et al. (2004) and Kannadhason et al. (2010) reported that the inclusion of DDGS decreased the expansion of extruded aquaculture feed. Smith and Aldrich (2023) reported that CFP resulted in decreased kibble diameter and SEI compared to SBM and CGM. However, kibble length and specific length were maintained among dietary treatments (Smith and Aldrich, 2023). In the current study, kibble diameter and SEI were lower for CFP vs. CON. Kibble length and specific length were higher for CFP vs. CON.

Previous research has reported that kibble expansion has an impact on hardness and compression energy (Moraru and Kokini, 2003; Yanniotis et al., 2007). The results for kibble hardness and toughness are interesting, as the diet with the least amount of expansion (BDY+DDGS) would be expected to result in greatest hardness and toughness. However, CFP resulted in the greatest kibble hardness and toughness in the current study. In contrast, Smith and Aldrich (2023) reported that a 25% inclusion of CFP did not impact kibble hardness or toughness when compared to SBM or CGM. This could indicate that differences in processing parameters and ingredient matrices can impact the hardness and toughness of an extruded kibble.

It is important to note that the extruder operator may have a significant impact on processing conditions and subsequent product characteristics. Therefore, it is difficult to state any significant conclusions on the impact of ingredient matrices on final product characteristics. Moving forward, it would be beneficial to maintain all input processing conditions or bulk density of treatments to better understand the effect of experimental ingredients.

### Diet chemical analyses

Dry matter and organic matter contents of dietary treatments were maintained at averages of 95 and 91%, respectively (Table 3). The average crude protein content of CON and BDY was 41% whereas the average crude protein content for BDY + DDGS and CFP was 38%. The fat content was greatest for BDY + DDGS at 14% compared to the remaining treatments at 13%. The BDY + DDGS treatment resulted in the highest total dietary fiber at 18% followed by CFP at 15% then CON and BDY at 13%. The same pattern among dietary treatments was observed with insoluble dietary fiber content. However, CFP contained the lowest amount of soluble dietary fiber among dietary treatments. Gross energy was comparable among dietary treatments at an average of 5000 kcal/kg.

**Table 3.** Analyzed chemical composition of feline diets containing yeast and ethanol co-products on a dry matter basis

	Treatment^1^			
Nutrient	CON	BDY	BDY + DDGS	CFP
Dry matter, %	95.03	95.71	94.38	95.50
Moisture, %	4.97	4.29	5.62	4.50
Organic matter, %	90.03	90.17	90.31	91.49
Ash, %	9.97	9.83	9.69	8.51
Crude protein, %	41.59	40.58	38.50	37.22
Crude fat, %	12.80	13.32	14.45	13.40
Total dietary fiber, %	13.66	13.19	18.46	15.05
Insoluble dietary fiber, %	10.09	10.04	14.34	12.40
Soluble dietary fiber, %	3.67	3.14	4.13	2.64
Gross energy, kcal/kg	4951.82	4997.76	5065.36	5001.77

^1^CON, control; BDY, brewer’s dried yeast; BDY+DDGS, brewer’s dried yeast and distillers dried grains with solubles; CFP, corn fermented protein.

The maintenance of moisture content among dietary treatments was expected as drying conditions were controlled during processing. The decreased protein content in BDY + DDGS was also expected as DDGS contained the least amount of protein among experimental ingredients at 32% (Kilburn-Kappeler et al., 2023). However, the decreased protein content of CFP was surprising as CFP contains 53% protein which is comparable to SBM (53%) and greater than BDY (47%). The protein content of dietary treatments could have been impacted by the CGM and/or corn starch that was added to balance the 20% inclusion of experimental ingredients. The increased fat content in BDY + DDGS compared to the other treatments was expected as DDGS had the greatest fat content among experimental ingredients. In addition, DDGS had the greatest amount of total dietary fiber at 45% followed by CFP at 35% which can explain the increased fiber content in the BDY+DDGS and CFP treatments.

### Feed intake and fecal characteristics

Food intake of cats fed dietary treatments was greater (*P* < 0.05) for BDY+DDGS at 81 g/d compared to BDY at 78 g/d (Table 4). Food intake for CON and CFP was intermediate at an average of 80 g/d. Wet fecal output of cats was greatest (*P* < 0.05) for BDY + DDGS at 59 g/d compared to the remaining treatments at an average of 49 g/d (Table 4). Fecal dry matter increased (*P* < 0.05) for cats fed BDY at 35% compared to cats fed CFP and CON (average, 32%). Fecal dry matter of cats fed BDY + DDGS was intermediate to cats fed BDY and CFP at 34%. Dry fecal output of cats was greatest (*P* < 0.05) for BDY + DDGS at 20 g/d compared to the remaining treatments at an average of 16 g/d. The number of defecations per day was greater (*P* < 0.05) for cats fed BDY + DDGS compared to cats fed CON and CFP, with BDY intermediate. Feces were firmer (*P* < 0.05) when cats were fed BDY + DDGS compared to CON, whereas fecal scores of cats fed BDY and CFP were intermediate. Fecal pH of cats was comparable (*P* > 0.05) among all dietary treatments.

**Table 4.** Food intake and stool quality parameters of cats fed diets containing yeast and ethanol co-products^1^

	Treatment^2^					
Parameter	CON	BDY	BDY+DDGS	CFP	SEM	*P*-value
Food intake, g/d	80.71^a,b^	77.90^b^	80.97^a^	78.90^a,b^	1.055	0.0186
Wet fecal output, g/d	51.80^b^	46.49^b^	59.22^a^	49.54^b^	2.316	<0.0001
Fecal dry matter, %	31.76^c^	35.29^a^	34.38^a,b^	32.91^b,c^	0.709	0.0002
Dry fecal output, g/d	16.36^b^	16.29^b^	20.24^a^	16.26^b^	0.585	<0.0001
Defecations per day	0.95^b^	0.96^a,b^	1.11^a^	0.89^b^	0.055	0.0030
Fecal score^3^	3.57^b^	3.73^a,b^	3.85^a^	3.77^a,b^	0.076	0.0095
Fecal pH	5.76	5.70	5.59	5.53	0.089	0.0645

^1^A total of 11 cats were enrolled in an incomplete 4 × 4 replicated Latin square design, resulting in 11 observations per treatment for each parameter.

^2^CON, control; BDY, brewer’s dried yeast; BDY + DDGS, brewer’s dried yeast and distillers dried grains with solubles; CFP, corn fermented protein.

^3^Scored on a 1 (liquid diarrhea) to 5 (dry hard pellets) scale with a score of 3.5 to 4.0 considered ideal.

^a-c^Means within a row lacking a common superscript letter are different (*P* < 0.05).

The minimal differences (<3g) in food intake are not of practical concern as they are unlikely to affect stool quality or nutrient digestibility. In agreement with the current study, Kilburn-Kappeler et al. (2023) reported similar fecal output, defecations per day, fecal score, and fecal pH of dogs fed BDY compared to CFP. Previous studies have reported differences in stool quality when comparing yeast to traditional ingredients. For example, Reilly et al. (2021) reported increased fecal output of dogs consuming dried yeast compared to poultry by-product meal. Whereas Holt and Aldrich (2022) reported that fecal output on a dry matter basis of cats fed Torula yeast was comparable to cats fed chicken meal and SBM. However, defecations per day of cats were greater when fed a diet containing SBM than a diet containing Torula yeast, with defecations of cats consuming chicken meal intermediate (Holt et al., 2022). Even with the differences in fecal output, Reilly et al. (2021) reported ideal fecal scores with no differences between dogs fed diets containing dried yeast or poultry by-product meal. Holt and Aldrich (2022), however, reported a greater frequency of softer feces when cats were fed Torula yeast. Both studies also reported that fecal pH was not impacted by test ingredients (Reilly et al., 2021; Holt and Aldrich, 2022). The fecal dry matter percent of dogs fed CFP and BDY was comparable (Kilburn-Kappeler et al., 2023) whereas feces were dryer for cats fed BDY compared to CFP in the current study. Holt and Aldrich (2022) reported that consumption of Torula yeast decreased the fecal dry matter percent of cats compared to SBM with no difference when compared to chicken meal. Based on the various results among the current and previous studies, it would be interesting to compare the stool quality of cats fed BDY and CFP to an animal-based ingredient. However, the similar stool quality of cats fed BDY and CFP in the current study indicates that the greater dietary fiber content of CFP did not significantly impact stool quality when compared to BDY, which is supported by the previous study completed in dogs (Kilburn-Kappeler et al., 2023).

### Apparent total tract digestibility

There were significant differences in dry matter digestibility among all dietary treatments (Table 5). The dry matter digestibility was greatest (*P* < 0.05) for CON at 82% and lowest (*P* < 0.05) for BDY+DDGS at 75%. The dry matter digestibility of BDY and CFP were intermediate at 81 and 79%, respectively. Organic matter digestibility was greatest (*P* < 0.05) for CON and BDY at an average of 87% and lowest (*P* < 0.05) for BDY+DDGS at 81%, with CFP being intermediate at 84%. Crude protein digestibility was highest (*P* < 0.05) for CON at 89% and lowest (*P* < 0.05) for BDY + DDGS and CFP at an average of 84% with BDY being intermediate at 87%. Fat digestibility was greater (*P* < 0.05) for CON at 96% compared to BDY + DDGS and CFP at 92 and 95%, respectively. In addition, fat digestibility was greater (*P* < 0.05) for BDY (96%) and CFP compared to BDY+DDGS. Total dietary fiber digestibility was greater (*P* < 0.05) for CON and BDY compared to BDY+DDGS and CFP. The digestibility of gross energy was greatest (*P* < 0.05) for CON and BDY at an average of 87% and lowest (*P* < 0.05) for BDY+DDGS at 82%, CFP was intermediate at 84%.

**Table 5.** Apparent total tract digestibility of diets containing yeast and ethanol co-products estimated by titanium dioxide as a dietary marker on a dry matter basis^1^

	Treatment^2^					
Nutrient, %	CON	BDY	BDY + DDGS	CFP	SEM	*P*-value
Dry matter	82.29^a^	80.94^b^	75.37^d^	78.72^c^	0.513	<0.0001
Organic matter	87.47^a^	87.08^a^	80.60^c^	83.61^b^	0.423	<0.0001
Crude protein	88.91^a^	86.99^b^	84.44^c^	84.51^c^	0.483	<0.0001
Crude fat	96.12^a^	95.64^a,b^	91.83^c^	94.97^b^	0.342	<0.0001
Total dietary fiber	61.20^a^	62.07^a^	49.47^b^	51.29^b^	1.208	<0.0001
Gross energy	87.76^a^	87.19^a^	81.53^c^	83.81^b^	0.430	<0.0001

^1^A total of 11 cats were enrolled in an incomplete 4 × 4 replicated Latin square design, resulting in 11 observations per treatment for each parameter.

^2^BDY, brewer’s dried yeast; BDY + DDGS, brewer’s dried yeast and distillers dried grains with solubles; CFP, corn fermented protein; CON, control.

^a-d^Means within a row lacking a common superscript letter are different (*P* < 0.05).

In agreement with the current study, Kilburn-Kappeler et al. (2023) reported increased dry matter, organic matter, crude protein, total dietary fiber, and gross energy digestibility of BDY compared to CFP when fed to dogs. In addition, fat digestibility of BDY and CFP was comparable in dogs (Kilburn-Kappeler et al., 2023). The decreased nutrient digestibility of the CFP treatment compared to the BDY treatment is likely due to the increased dietary fiber content of CFP. As increased dietary fiber has been known to decrease nutrient digestibility in both dogs and cats (Burrows et al., 1982; Fahey et al., 1990; Sunvold et al., 1995; Fischer et al., 2012). The TDF digestibility in the current study was higher than expected however values are similar to those reported for diets containing SBM and yeast fed to cats (Holt and Aldrich, 2022). In addition, previous studies have reported TDF digestibility of diets containing 15% to 25% CFP to be high at an average of 46.5% when fed to both dogs and cats (Kilburn-Kappeler et al., 2022, 2023; Kilburn-Kappeler and Aldrich, 2023; Smith and Aldrich, 2023). Previous studies have reported a decrease in dry matter, organic matter, and fat digestibility of a diet containing yeast compared to a diet containing an animal-based protein source in dogs and cats (Reilley et al., 2021; Holt and Aldrich, 2022). Whereas the digestibility of crude protein and total dietary fiber was comparable among the two diets in both studies (Reilly et al., 2021; Holt and Aldrich, 2022). Therefore, it would be interesting to compare the digestibility of BDY and CFP to an animal-based ingredient when fed to dogs and cats.

### Fecal chemical analysis

Total SCFA concentrations in fecal samples of cats fed dietary treatments ranged from 516 to 545 μmol/g (Table 6). However, no significant differences in total SCFA were observed among dietary treatments. There were also no differences (*P* > 0.05) in percent acetate, propionate, or butyrate among dietary treatments with averages of 73%, 21%, and 7%, respectively. There were no significant differences observed in total BCFA concentrations among dietary treatments, ranging from 30 to 35 μmol/g. For BCFA, percent isovalerate, isobutyrate, and valerate were also comparable (*P* > 0.05) among dietary treatments at averages of 27%, 16%, and 57%, respectively.

**Table 6.** Chemical analysis of feces from cats fed diets containing yeast and ethanol co-products^1^

	Treatment^2^					
Parameter	CON	BDY	BDY + DDGS	CFP	SEM	*P*-value
Total SCFA^3^, μmol/g DM feces	545.00	515.90	538.57	524.64	62.3286	0.9653
Acetate, %	75.33	72.25	71.56	71.72	2.0013	0.2151
Propionate, %	18.40	20.57	21.79	21.31	1.6787	0.2139
Butyrate, %	6.26	7.19	6.64	6.98	0.6029	0.4465
Total BCFA^4^, μmol/g DM feces	33.23	35.19	30.09	31.17	4.8187	0.7263
Isovalerate, %	26.74	27.28	26.78	28.12	1.4172	0.7468
Isobutyrate, %	17.52	14.45	16.33	13.96	2.7396	0.5414
Valerate, %	55.74	58.27	56.89	57.92	2.7313	0.7903

^1^A total of 11 cats were enrolled in an incomplete 4 × 4 replicated Latin square design, resulting in 11 observations per treatment for each parameter.

^2^BDY, brewer’s dried yeast; BDY + DDGS, brewer’s dried yeast and distillers dried grains with solubles; CFP, corn fermented protein; CON, control.

^3^Total SCFA (acetate + propionate + butyrate); individual SCFA is expressed as a percent of total SCFA.

^4^Total BCFA (isovalerate + isobutyrate + valerate); individual BCFA is expressed as a percent of total BCFA.

In agreement with the current study, Kilburn-Kappeler et al. (2023) reported no significant differences in SCFA or BCFA concentrations in fecal samples of dogs fed BDY or CFP. These results indicate that CFP did not alter fermentation within the large intestine of dogs and cats when compared to BDY. Yet, Reilly et al. (2021) reported an increase in total SCFA as well as an increase in the proportion of acetate and butyrate in feces of dogs fed dried yeast compared to poultry by-product meal. However, the proportion of propionate in feces was comparable between the two diets (Reilly et al., 2021). In addition, total BCFA as well as the proportion of isobutyrate, isovalerate, and valerate were maintained in fecal samples of dogs fed diets containing dried yeast and poultry by-product meal (Reilly et al., 2021). Therefore, it appears that yeast may promote the beneficial production of SCFA compared to an animal-based ingredient. A future study would be warranted to determine if BDY and CFP have a similar effect when compared to an animal-based ingredient.

It is important to acknowledge that excreted fecal microbes may underestimate apparent nutrient digestibility if hindgut fermentation has been increased by dietary fiber levels and/or yeast cell wall components. However, similar levels of total fecal SCFA and BCFA across treatments do not support an increase in hindgut fermentation in this study. Nevertheless, accounting for microbial contributions in fecal nutrient excretion could provide more accurate estimates of dietary nutrient digestibility.

### Palatability

The palatability evaluation indicated no preference between the CON or BDY and BDY + DDGS treatments when fed to cats (Table 7), as first choice and intake ratios were comparable (*P* > 0.05) for each test. There was also no preference between CON or CFP based on the first choice of cats. However, cats consumed more CON compared to CFP which is indicated by the significant intake ratio.

**Table 7.** First choice (FC) and intake ratio (IR) of cats fed diets containing yeast and ethanol co-products^1^

Diet comparison (A vs. B)^2^	FC^3^	IR^4^
BDY vs. CON	21	0.591
BDY+DDGS vs. CON	23	0.350
CFP vs. CON	24	0.256^*^

^1^A total of 20 cats were fed each diet comparison over 2 d, resulting in 40 observations per comparison.

^2^BDY, brewer’s dried yeast; BDY + DDGS, brewer’s dried yeast and distillers dried grains with solubles; CFP, corn fermented protein; CON, control.

^3^Number of first visits to bowl A out of 40 observations.

^4^IR = intake (g) of diet A/total intake (g) of diets A + B.

^*^Comparison differs (*P* < 0.05).

Yeast products have been used as palatability enhancers in pet food for many years (Swanson and Fahey, 2007). The increased palatability of yeast is attributed to nucleotides and high glutamic acid concentration which provides the umami, or meaty, aroma and taste (Nagodawithana, 1992; Ugawa and Kuihara, 1994). However, palatability results are variable among studies, indicating that the type of yeast and inclusion level may impact results. Swanson and Fahey (2007) reported that a 1% inclusion of brewer’s yeast was more palatable than a 1% inclusion of corn-wet milling yeast when fed to both dogs and cats. In the current study, cats preferred the CON to CFP based on the intake ratio but had no preference when comparing BDY to CON. However, CFP was not directly compared to BDY which would have provided interesting results. Kilburn-Kappeler et al. (2023) reported that dogs had no preference between CON and BDY but preferred CON over CFP based on first choice and intake ratio. Based on the amino acid profile reported by Kilburn-Kappeler et al. (2023), CFP contains more glutamic acid compared to BDY (8.4% vs 5.3%, respectively). Therefore, it would be expected that CFP would have an intensified umami aroma and flavor, increasing palatability. However, these results indicate that distiller’s yeast may be less palatable for dogs and cats compared to brewer’s yeast. It is also important to consider the effects of processing and kibble texture on palatability (Koppel et al., 2015). As the CFP treatment resulted in the hardest and toughest kibble which may have caused decreased palatability. Regardless, cats willingly consumed all treatments and no refusals were observed during the digestibility study when choice was not a factor.

## Conclusion

Compared to BDY, CFP required adjustments in processing parameters to achieve a similar bulk density of kibble. However, even with the similar bulk density, CFP resulted in a harder and tougher kibble compared to BDY. Surprisingly, the increased fiber content in CFP compared to BDY did not impact stool quality or SCFA and BCFA concentrations in fecal samples of cats. However, it likely decreased the nutrient digestibility of CFP compared to BDY. In addition, BDY appeared to be more palatable than CFP when compared to the control containing SBM. However, BDY and CFP were not directly compared in the palatability assessment and cats willing consumed all diets in the digestibility trial. To classify or utilize CFP as a yeast source, further research is warranted to evaluate if the yeast component of CFP has any potential benefit to animal health.

## Abbreviations:

AAFCO: Association of American Feed Control Officials
AOAC: Association of Official Agricultural Chemists
ATTD: apparent total tract digestibility
BCFA: branched-chain fatty acid;
BDY: brewer’s dried yeast
BW: body weight
CFP: corn fermented protein
CGM: corn gluten meal
CON: control
DDGS: distillers dried grains with solubles
EX: extruder
MC: in-barrel moisture content
NRC: National Research Council
PC: preconditioner
SBM: soybean meal
SCFA: short-chain fatty acid
SEI: sectional expansion index
SME: specific mechanical energy
TiO_2_: titanium dioxide
TMF: total mass flow
